# Effects of Intermittent Theta Burst Preconditioning on 40Hz Gamma Wave Synchronization

**DOI:** 10.1002/alz.088567

**Published:** 2025-01-09

**Authors:** Donghyun Ahn, Sungwon Roh, Haram Lee, Haein Kim, Youngseok Lee, Jin Yong Jeon

**Affiliations:** ^1^ Hanyang University, Seoul, Seoul Korea, Republic of (South)

## Abstract

In the treatment of Alzheimer’s disease (AD), traditional 40Hz gamma wave stimulation, while effective in improving cognitive function, has been limited by the lengthy duration of treatment. To address this, our study sought to enhance the time efficiency of 40Hz stimulation by utilizing intermittent theta‐burst stimulation (iTBS) as a pretreatment. Experiments were conducted on AD mouse models, divided into four groups: no stimulation, 40Hz stimulation only, iTBS stimulation only, and combined iTBS and 40Hz stimulation. The groups were assessed for cognitive function and behavioral changes, as well as neuro‐pathological indicators.

The results revealed that the group receiving combined iTBS and 40Hz stimulation showed significant improvements in cognitive function and behavior from the fifth day of treatment, compared to the groups receiving only a single type of stimulation. This indicates that the combined stimulation is not only effective but also significantly reduces the treatment time, demonstrating rapid effects. Therefore, the combination of iTBS and 40Hz stimulation presents a new approach in AD treatment, enhancing both time efficiency and therapeutic effectiveness. These findings offer important implications for future clinical research and applications.

